# Dynamic functional connectivity changes associated with psychiatric traits and cognitive deficits in Cushing’s disease

**DOI:** 10.1038/s41398-023-02615-y

**Published:** 2023-10-05

**Authors:** Zhebin Feng, Haitao Zhang, Tao Zhou, Xinguang Yu, Yanyang Zhang, Xinyuan Yan

**Affiliations:** 1 Department of Neurosurgery, Chinese PLA General Hospital, Haidian District, Beijing, PR China; 2 Department of Respiratory Medicine, Anhui Provincial Children’s Hospital, Hefei, Anhui PR China; 3 Neurosurgery Institute, Chinese PLA General Hospital, Beijing, PR China; 4 Department of Psychiatry, University of Minnesota Medical School, Minneapolis, MN USA

**Keywords:** Diseases, Neuroscience

## Abstract

Cushing’s disease is a rare neuroendocrine disorder with excessive endogenous cortisol, impaired cognition, and psychiatric symptoms. Evidence from resting-state fMRI revealed the abnormalities of static brain connectivity in patients with Cushing’s disease (CD patients). However, it is unknown whether the CD patients’ dynamic functional connectivity would be abnormal and whether the dynamic features are associated with deficits in cognition and psychopathological symptoms. Here, we evaluated 50 patients with Cushing’s disease and 57 healthy participants by using resting-state fMRI and dynamic functional connectivity (dFNC) approach. We focused on the dynamic features of default mode network (DMN), salience network (SN), and central executive network (CEN) because these are binding sites for the cognitive-affective process, as well as vital in understanding the pathophysiology of psychiatric disorders. The dFNC was further clustered into four states by k-mean clustering. CD patients showed more dwell time in State 1 but less time in State 4. Intriguingly, group differences in dwell time in these two states can explain the cognitive deficits of CD patients. Moreover, the inter-network connections between DMN and SN and the engagement time in State 4 negatively correlated with anxiety and depression but positively correlated with cognitive performance. Finally, the classifier trained by the dynamic features of these networks successfully classified CD patients from healthy participants. Together, our study revealed the dynamic features of CD patients’ brains and found their associations with impaired cognition and emotional symptoms, which may open new avenues for understanding the cognitive and affective deficits induced by Cushing’s disease.

## Introduction

Cushing’s disease is characterized by excess endogenous cortisol secretion [1] and served as a unique and natural model for investigating the effects of elevated endogenous cortisol levels on brain functions and structure [2]. It is also a good model for unraveling the linkage between stress-related brain dysfunctions and psychiatric symptoms [3]. Long-term exposure to hypercortisolism negatively affects patients’ physical and mental health, such as depression, anxiety, and psychosis [1, 4], as well as shows deleterious effects on cognitive function including impaired executive function, working memory, and attention [5–7].

Research progress on Cushing’s disease, which depends on static resting-state fMRI, revealed that patients with Cushing’s disease showed increased functional connectivity between the default mode network (DMN) and left lateral occipital cortex [2], and hippocampus [8]. Cortisol increase would induce connectivity changes within the DMN and salience network (SN) [9], and the DMN’s activity correlated with the morning cortisol level of patients with Cushing’s disease [10].

Despite these advances leading to an improved understanding of Cushing’s disease, it remains enigmatic how the abnormal brain connectivity within large-scale networks and how the different brain networks interact would contribute to the deficits in impaired cognitive function, as well as psychopathological symptoms. Furthermore, recent years have witnessed an increasing number of studies providing solid evidence that the brain is a dynamic system rather than a static one on a micro-time scale [11, 12]. Dynamic functional connectivity (dFNC), which is implemented by the sliding window method [13], is an ideal approach to characterize the dynamic nature of brain [11], as well as detect and predict diseases [14, 15]. However, to our knowledge, no studies have ever investigated dynamic brain functional connectivity for patients with CD.

We focus here on dynamic functional connectivity and emphasize the role of default mode network (DMN), salience network (SN), and central executive network (CEN). These large-scale neurocognitive networks are critical for cognitive and affective processing [16] and are highly related to stress and cortisol level. Deficits or abnormal connectivity within these three networks are associated with a wide range of stress-related psychiatric disorders [17], as well as the high level of cortisol production [18, 19]. For example, the network-connectivity changes between SN and DMN [20, 21], SN and CEN [22] corresponded to increased cortisol levels. Furthermore, our previous studies also identified that CD patients would show dysregulations of resting-state functional connectivity patterns with DMN [10, 23]. Since CD patients also suffer from cognitive impairment and neuropsychological symptoms, including depression and anxiety, which DMN, SN, and CEN mainly modulate, we hypothesized that these three networks are critical to understanding Cushing’s disease and its comorbidity. Here we aimed to investigate two research questions. First, whether there are group differences (CD patients vs. healthy controls) in the dynamic functional connectivity within DMN, SN, and CEN; second, whether the differences can explain the psychiatric symptoms and cognitive impairments in CD patients.

We configure our design with a sliding-window approach [11, 13] to portray the features of dynamic functional connectivity (dFNC) within DMN, SN, and CEN among patients with Cushing’s disease (*N* = 50) and healthy controls (*N* = 57). We first compared the temporal properties between healthy and CD patients. Then we conducted correlation and mediation analysis to see whether and how the differences in dFNC would contribute to patients’ psychiatric and physiological symptoms and cognitive deficits. We finally implemented a classification machine learning algorithm based on dynamic FNC features within these three networks to see whether these dynamic features would identity CD patients successfully.

## Materials and methods

### Ethic approval

The experimental protocol was in accordance with principles of the Declaration of Helsinki and approved by a local research ethics Committee of The First Medical Center of Chinese PLA General Hospital (Beijing, China). All participants provided written informed consent after the experimental procedure had been fully explained and were reminded of their right to withdraw at any time during the study.

### Participants

The current study recruited 50 patients with Cushing’s disease (CD patients) and 57 healthy controls (HC) who were matched in age, gender, and education (Table 1). The CD patients were recruited from the Department of Neurosurgery, The First Medical Center of Chinese PLA General Hospital, between May 2017 and November 2019. The following criteria confirmed Cushing’s disease and its etiology: clinical features (e.g., moon face, supraclavicular fat pad, truncal obesity), elevated 24-h urinary free cortisol (24-h UFC, reference range 98.0–500.1 nmol/24 h), absence of normal cortisol circadian rhythm, elevated ACTH levels (reference range at 0800 h: <10.12 pmol/L), elevated cortisol secretion rates (reference range of cortisol level at 0800 h, 198.7–797.5 nmol/L), absence of normal suppression in midnight (1 mg) dexamethasone suppression test and low dose (2 mg) dexamethasone suppression test (but >50% suppression with a high dose (8 mg) of dexamethasone), and a central to peripheral ACTH ratio >2 for petrosal sinus sampling and pathology after surgery. Healthy controls (HC) were recruited from the local community through poster advertisements and were interviewed by experienced psychiatrists to ensure the absence of current or history of any mental disorder. Demographic information and clinical characteristics of all CD patients and healthy controls were shown in Table 1.

**Table 1:** Demographic and clinical data from healthy controls and CD patients.

		Healthy controls (*N* = 57)		CD patients (*N* = 50)		Healthy controls vs. CD patients (*p*-value)
		Mean (SD)	Min-Max	Mean (SD)	Min-Max	
Age(year)		35.421(11.098)	20–63	37.960(10.728)	15–62	0.233
Education(year)		12.350(3.286)	5–17	11.040(4.015)	3–18	0.070
Gender(m/f)		0/57	–	0/50	–	–
Disease duration(month)		–	–	43.370(53.698)	1–300	–
Plasma cortisol level (nmol/L)	00:00	–	–	603.612(226.104)	290.420–11179.600	–
	08:00	–	–	710.909(280.709)	221.440–1691.400	–
	16:00	–	–	638.121(236.342)	208.990–1534.740	–
ACTH (nmol/L)	00:00	–	–	14.159(7.655)	3.640–40.200	–
	08:00	–	–	18.644(15.443)	2.310–72.400	–
	16:00	–	–	17.498(10.850)	3.220–51.100	–
UFC (nmol/24 h)		–	–	2156.4372(1409.0234)	423.500–6777.400	–
Cushing QOL		–	–	36.617(8.894)	20–55	–
SDS		32.000(3.533)	25–42	49.425(12.379)	27–76	<0.001
SAS		24.631(3.642)	20–34	37.893(7.777)	24–59	<0.001
CNPI		–	–	11.000(10.836)	0–46	–
MoCA-BJ	Visuospatial/Executive	4.777(0.540)	3–5	3.173(1.141)	0–5	<0.001
	Naming	2.777(0.421)	2–3	2.326(0.844)	0–3	0.002
	Memory	4.305(0.920)	2–5	2.565(1.544)	0–5	<0.001
	Attention	5.833(0.447)	4–6	5.087(1.170)	0–6	<0.001
	Language	2.472(0.608)	1–3	2.000(0.788)	0–3	0.004
	Abstraction	1.722(0.566)	0–2	1.369(0.770)	0–2	0.019
	Orientation	6.000(0)	6–6	5.804(0.542)	4–6	0.018
MoCA-BJ total score		27.889(2.135)	21–30	22.369(4.428)	8–29	<0.001

Independent T test was adopted to test between-group difference (HC vs. CD).

*Cushing QOL* Cushing quality of life, *SDS* Self-Rating Depression Scale, *SAS* Self-Rating Anxiety Scale, *CNPI* Chinese version of neuropsychiatric inventory, *MoCA-BJ* Montreal Cognitive Assessment-Beijing Version.

### Clinical data acquisition, neuropsychological and neuropsychiatric assessment

Biometric measurements of the CD patients, including 24-h urinary free cortisol (UFC) levels, plasma Cortisol level (at 0000 h, 0800 h, 1600 h) and adrenocorticotropin (ACTH) level (at 0000 h, 0800 h, 1600 h) from a peripheral vein. Clinical severity of CD patients was obtained using the Cushing Quality of Life Scale (Cushing QOL) [24]. We also included the neuropsychological and neuropsychiatric assessments such as Self-Rating Depression Scale (SDS) [25], Self-Rating Anxiety Scale (SAS) [26], Montreal Cognitive Assessment-Beijing Version (MoCA-BJ) [27], and Chinese version of neuropsychiatric inventory (CNPI) [28].

### Image acquisition

Functional brain images were acquired using a 3-Tesla GE750 scanner at the First Medical Center of Chinese PLA General Hospital (Beijing, China). Blood oxygen level-dependent (BOLD) gradient echo planar images (EPIs) were obtained using an 8-channel head coil [64 × 64 × 36 matrix with 3.5 × 3.5 × 3.5 mm spatial resolution, repetition time (TR) = 2000 ms, echo time (TE) = 30 ms, flip angle = 90°, field of view (FOV) = 256 × 256 mm^2^]. A high-resolution T1-weighted structural image (256 × 256 × 144 matrix with a spatial resolution of 1 × 1 × 1 mm, repetition time (TR) = 6700 ms, echo time (TE) = 29 ms, flip angle = 7°) was subsequently acquired. During scanning, all participants were fitted with soft earplugs, and were requested to keep their eyes closed, to stay awake and not to think of anything.

### Data preprocessing

The fMRI data was preprocessed using SPM12 (Wellcome Trust Centre for Neuroimaging, London). The first 10 volume of the functional images were discarded to avoid initial steady-state problems. Then functional images were spatially realigned to the first image for motion correction and corrected for slice acquisition temporal delay. Subsequently, functional images were co-registered to each participant’s segmented gray matter T1 image, then spatially normalized to the Montreal Neurological Institute (MNI) coordinate system, resampled to 3 × 3 × 3 mm voxels. Finally, all functional images were spatially smoothed with an isotropic 4 mm FWHM Gaussian kernel.

### Group ICA and post-processing

**Fig. 1:**
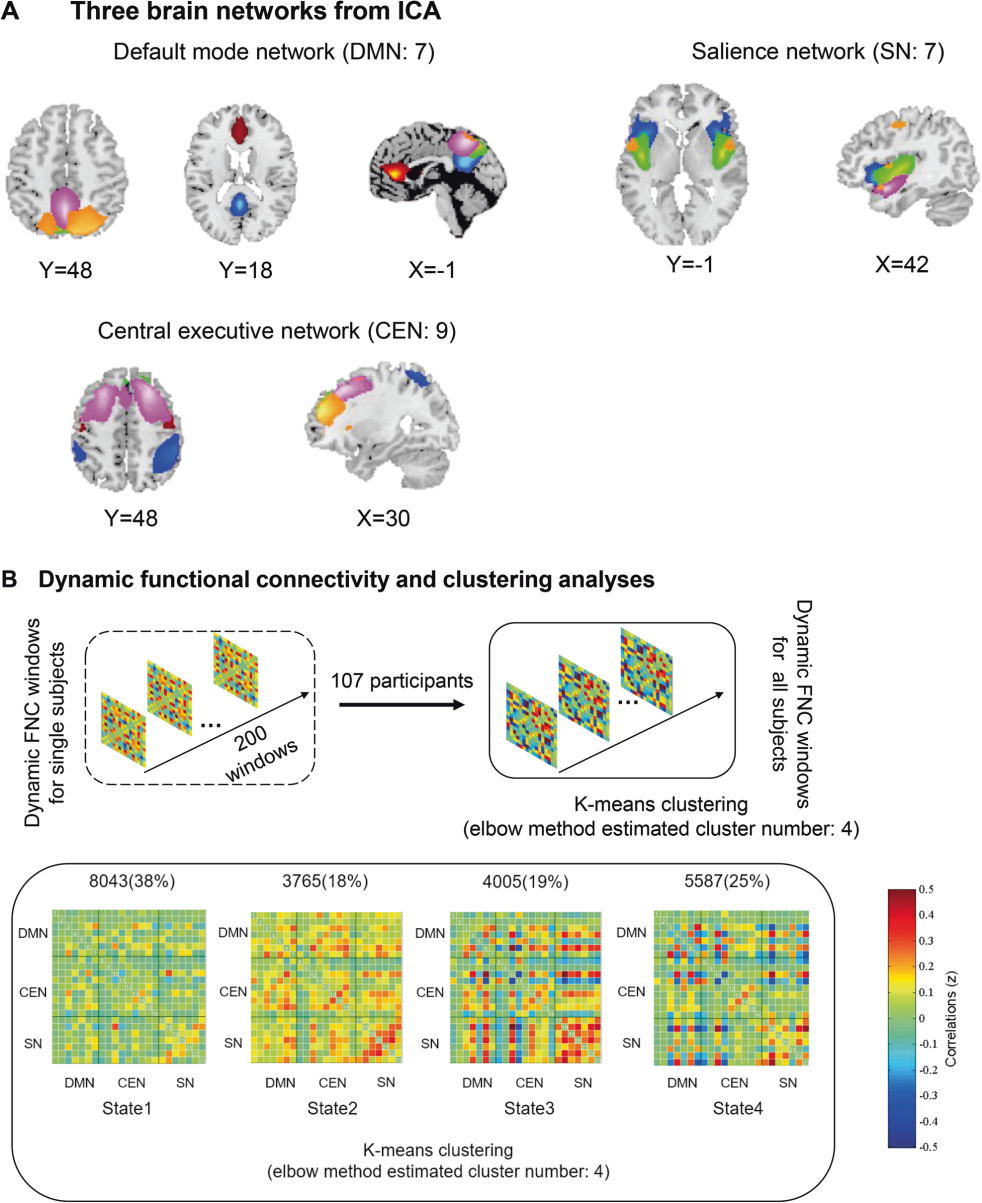
Composite map of the three networks. And the pipeline of dynamic functional connectivity and clustering analyses. **A** The three brain networks, default mode network (DMN, including 7 components), central executive network (CEN, including 9 components) and salience network (SN, including 7 components) are derived from group spatial independent components analyses among all participants. **B** First, for each participant, the dynamic functional connectivity (FNC) matrices are estimated on each sliding window (200 windows) of a set of components within the three networks. Then we applied k-means clustering algorithm on the dynamic FNC matrices across all subjects to assess the reoccurring FNC’s states. Optimal number of states was determined by elbow method. We showed the averaged FNC pattern and the corresponding total number of windows in each state, percentage of each occurrence was presented in parentheses. The color bar represents the z value of FNC.

Preprocessed data were decomposed into functional components that exhibited a unique time course profile using the group-level spatial independent component analysis, which was implemented in the GIFT toolbox (http://mialab.mrn.org/software/gift/) [29]. First, a subject-specific data reduction principal component analysis (PCA) was performed in which 120 principal components remained. Then at group level, we adopted a high model order ICA to reduce the resting state data into 100 group independent components [30] using the expectation-maximization (EM) algorithm [31] in GIFT. Further, the Infomax ICA algorithm in ICASSO [32] was repeated 20 times [33] to ensure the reliability and stability. Subject-specific spatial maps and time-courses were estimated using the back-reconstruction approach (GICA) [34]. We characterized 50 components as intrinsic connectivity networks (ICNs) by applying the following criteria:[13, 35] whether the peak activation coordinates of the functional components were primarily located in gray matter, and with minimal spatial overlap with white matter structures, vascular, ventricular, edge regions corresponding to artefacts, and susceptibility artifacts. We sorted these 50 meaningful independent components into the interested functional networks including: default mode network (DMN), central executive network (CEN) and salience network (SN) (Fig. 1) according to the spatial correlation values between independent components and the given template [36]. Additional post-processing was conducted to remove remaining noise. Time-courses of the seven components were detrended, despiked and low-pass filtered with a high-frequency cutoff of 0.15 Hz [13]. Moreover, we regressed out the six parameters of head movement.

### Dynamic functional connectivity

Sliding window approach is the most common way to investigate the nonstationary nature of functional connectivity (FC) of fMRI data. We conducted dynamic FC analysis using the DFC network toolbox in GIFT. In line with previous studies [13, 36], a window of 60 s width (30 TR), sliding in steps of one repetition time was applied to divide the time-courses of each independent components into 200 windows. As covariance estimation using time series of shorter length can be noisy, the regularized inverse covariance matrix (ICOV) was adopted [37]. Following graphic LASSO framework [38], we imposed an additional L1 norm of the precision matrix to enforce sparsity.

### Clustering analysis

Based on previous studies, we applied a k-means clustering algorithm on windowed functional connectivity matrices [39] to assess the frequency and structure of reoccurring functional connectivity patterns (states) across all subjects. We used Manhattan distance function to estimate the similarity between different time windows of FC matrices, which had been demonstrated as an effective measure for high-dimensional data [40]. To obtain the optimal number of states, a cluster validity analysis (silhouette) was conducted on the exemplars of all the subjects. To avoid cost function convergence to the local optimal solution, all clustering analyses were iterated 5 times in GIFT, and the best result was used. Finally, we determined the optimal number of clusters as equal to four (*k* = 4). According to the clustering results, three temporal properties of dynamic FC states derived from each subject’s state vector were calculated: (i) mean dwell time, measured as the average number of consecutive windows belonging to one state; (ii) fraction of time, measured as the proportions of total windows in one state; (iii) number of transitions, defined as the number of state transitions during the entire scan.

### Mediation analyses

Bootstrapping method was used to estimate the mediation effect. Bootstrapping is a nonparametric approach to effect-size estimation and hypothesis testing that is increasingly recommended for many types of analyses, including mediation [41, 42]. Bootstrapping generates an empirical approximation of the sampling distribution of a statistic by repeated random resampling from the available data and uses this distribution to calculate *p*-values and construct confidence intervals (5000 resamples were taken for these analyses). Moreover, this procedure supplies superior confidence intervals (CIs) that are bias-corrected and accelerated [43, 44].

### Classification analyses using dynamic functional connectivity

We conducted classification analyses based on dynamic FNC features [35] to classify each kind of patients. Specifically, we firstly formed a regression matrix, R_groups × cluster centroids_, then regressed out the windowed FNC matrices at each time window using the regression matrix for each participant. These analyses end up with eight β coefficients for each time window for each participant. Next, we computed the mean β coefficients for all time windows. Thus, we got eight mean β coefficients for each participant. These mean β coefficients served as the dynamic FNC features for the classification analysis. The classification analysis using supervised machine learning method, linear support vector machine algorithm (http://www.csie.ntu.edu.tw/~cjlin/libsvm/) with a standard 10-fold cross-validation. We randomly divided the data into 10 subgroups, used the trained classifier from the nine subgroups to predict the performance on the left one subgroup, and repeated the procedure for 100 times. We reported the averaged classification accuracy for each group across these 100 times.

## Results

### Neuropsychological and neuropsychiatric difference between healthy controls and CD patients

Patients with Cushing’s disease reported higher depression, anxiety, and higher frequency and severity mental illness than healthy controls. Additionally, CD patients also behaved impaired cognitive ability than healthy controls (see Table 1)

### Functional connectivity within DMN, CEN and SN networks in the four states

Spatial map of default mode network, central executive network and salience network identified using the group independent component analysis was shown in Fig. 1A. Independent components were grouped based on their anatomical and presumed functional properties: default mode network (ICs, 9, 12, 27, 28, 32, 44, 74), central executive network (ICs, 15, 21, 26, 48, 50, 63, 85, 89, 97), and salience network (ICs, 20, 43, 57, 59, 76, 82, 92). We adopted a k-means clustering algorithm on the dynamic functional connectivity (dFNC) from all subjects into four connectivity states. Figure 1B shows the cluster centroid and the percentage of occurrences of each state (arranged in the order of emergence).

### Different temporal properties between HC and CD patients

**Fig. 2:**
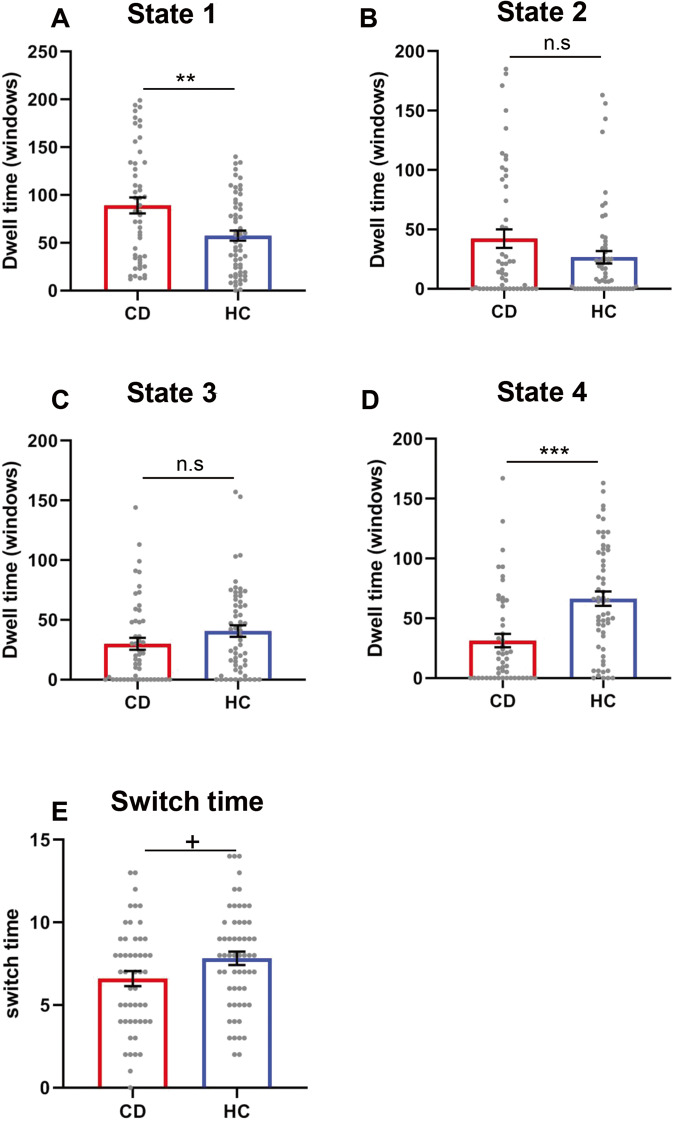
Mean dwell time of dynamic FNC states and number of transitions between CD patients and healthy controls. **A** In State 1, CD patients engaged higher mean dwell time than healthy control did. **B**, **C** In State 2 and State 3, no difference was found between CD patients and healthy controls. **D** In State 4, CD patients showed significant less mean dwell time than healthy controls. **E** There was marginally significant difference (after FDR correction) on number of transitions between CD patients and healthy controls. Multiple comparisons were corrected by FDR, *p* < 0.05 (Error bars represent standard error. *p* < 0.01**, *p* < 0.001***, *p* < 0.08^+^, N.S not significant). HC Healthy controls, CD patients with Cushing’s disease.

We firstly compared the mean dwell time between healthy controls and CD patients in each state (Fig. 2A–D). Using independent T test, we found that the CD patients had higher mean dwell time than HC in State 1 (CD patients: 89.040 ± 59.216 vs. HC: 57.491 ± 40.671; *t*(105) = 3.244, *p* = 0.002), but less mean dwell time than HC in State 4 (CD patients: 31.300 ± 39.413 vs. HC: 66.438 ± 45.734; *t*(105) = −4.227, *p* < 0.001). We did not observe significant difference in State 2 (CD patients vs. HC: *t*(105) = 1.700, *p* = 0.092), nor in State3 (CD patients vs. HC: *t*(105) = −1.517, *p* = 0.132). For the switch time (i.e., the number of transitions), CD patients revealed less transition number than healthy controls did (CD patients: 6.600 ± 3.187 vs. HC: 7.824 ± 3.059; *t*(105) = −2.205, *p* = 0.045; Fig. 2E). Multiple comparisons were corrected by false-discovery rate (FDR), *p* < 0.05. All contrasts remained the same after FDR correction excepted the results of switch time became marginally significant, FDR corrected *p* = 0.075. Group difference on fraction of time in each state was similar with the mean dwell time (see Supplementary Table S1). Levene’s test is used to check that variances are equal for all samples.

### Correlation between dynamic FNC properties and clinical characteristics

To examined whether the dynamic FNC properties were associated with clinical characteristics, we did Pearson correlation analyses. Since the group differences were found in State 1 and State 4, we only restricted our analyses on these two states. Notably, we found that the dwell time in State 1 positively correlated with the self-reported anxiety (SAS), and cortisol level at 8:00, 16:00, 00:00, ACTH at 8:00, 16:00, as well as elevated 24-h urinary free cortisol. That is, the longer time spent on State 1 which with more sparsely connected pattern, the worse the mental health and higher cortisol level. We also detected a robust negative correlation between dwell time of State 1 and global cognitive scales (MoCA), which indicated that more time spent in State 1, the worse cognitive ability would be. In the contrary, dwell time in State 4 showed significant negative correlation with the self-reported depression, anxiety, and cortisol level at 8:00, 16:00, 00:00. More dwell time in State 4 predicted better cognitive performance measured by MoCA (all results see Table 2). Multiple comparisons were conducted by FDR, *p* < 0.05.

**Table 2:** Correlations between dynamic functional connectivity temporal properties in cognitive control network and clinical data.

		Cushing-QOL^a^	SDS	SAS	CNPI^a^	MoCA-BJ	Cortisol 0000^a^	Cortisol 0800^a^	Cortisol 1600^a^	ACTH 0000^a^	ACTH 0800^a^	ACTH 1600^a^	UFC^a^
Dwell time State 1	*r*	−0.015	0.210	0.238	0.219	−0.533	0.293	0.298	0.399	0.232	0.294	0.347	0.413
	*P*-value	0.919	0.054	0.028	0.126	**<0.001**	0.043	0.036	**0.005**	0.117	0.038	**0.015**	**0.006**
Dwell time State 4	*r*	0.034	−0.367	−0.323	−0.038	0.452	−0.362	−0.404	−0.322	−0.218	−0.267	−0.209	−0.266
	*P*-value	0.823	**0.001**	**0.003**	0.794	**<0.001**	**0.011**	**0.004**	**0.024**	0.141	0.061	0.149	0.084

Significant results which were survived after FDR (*p* < 0.05) correction were reported in bold type.

*Cushing QOL* Cushing quality of life, *SDS* Self-Rating Depression Scale, *SAS* Self-Rating Anxiety Scale, *CNPI* Chinese version of neuropsychiatric inventory, *MoCA-BJ* Montreal Cognitive Assessment-Beijing Version.

^a^Correlation performed for the CD patients (*N* = 50).

### Dwell time in State 1 and State 4 within cognitive control network mediate group difference in cognitive performance

**Fig. 3:**
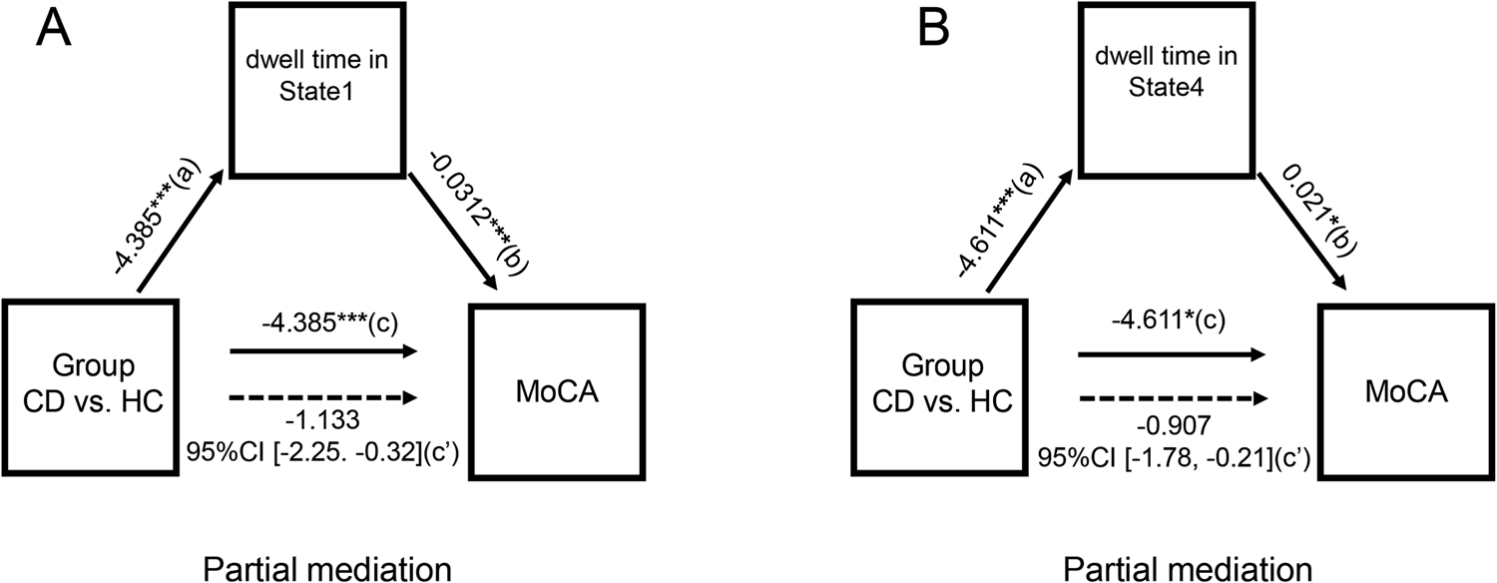
Mediation effect of dwell time in State 1 and State 4 on group difference on cognitive performance. **A** Dwell time in State 1 and **B** dwell time in State 4 significant partially mediated the difference between CD patients and healthy controls on cognitive performance measured by MoCA. HC Healthy controls, CD patients with Cushing’s disease.

Interestingly, we found the dwell time in State 1 and State 4 significantly mediated the difference between individuals with excessive high cortisol level (CD patients) and healthy controls on cognitive performance. That is, lower cognitive performance in CD patients was linked with more dwell time in State 1 (Fig. 3A), and less dwell time in State 4 (Fig. 3B) within the three networks.

### Distinct network-based functional connectivity between CD patients and healthy controls and its associations with psychiatric symptoms and cognitive performance

We have already known that the difference on dwell time in State 1 and State 4 can explain the group difference (i.e., CD patients vs. healthy controls) on cognitive performance. We further characterized the State 1 and State 4 by analyzing functional connectivity between the three networks, as well as the functional connectivity within each network. Results showed that in State 1, the CD patients had weaker connectivity within DMN (t(104)^1^ = −2.584, *p* = 0.011), and the connections between CEN and DMN (*t*(104) = −5.141, *p* < 0.001), CEN and SN (*t*(104) = −4.732, *p* < 0.001) were also weaker than healthy controls. And in State 4, CD patients showed weaker functional connections between DMN and SN (*t* (84)^2^ = −4.203, *p* < 0.001), as well as DMN and CEN (*t*(84) = −3.547, *p* = 0.001). Moreover, in State 4, functional connection between DMN and SN was negatively correlated with anxiety level measured by SAS (*r*(68) = −0.336, *p* = 0.005), and depression level measured by SDS (*r*(68) = −0.320, *p* = 0.008), but positively correlated with cognitive performance measured by MoCA (*r*(65) = 0.421, *p* < 0.001). Since CD patients showed decreased connection between DMN and SN, these results may suggest that the connection between DMN and SN was critical for understanding the psychiatric symptoms and cognitive deficits in CD patients. All significant results reported here were survived after FDR (*p* < 0.05) correction.

We did not find significant associations between functional connectivity of neither inter-network and intra-network and psychiatric symptoms and cognitive deficits in State 1. No significant correlation results were found between the inter-network and intra-network connectivity and physiological indices (i.e., cortisol, ACTH, and UFC) in these two states, which may suggest that the dwell time in specific state would be more sensitive to physiological change.

### Classification results based on dynamic FNC features

**Fig. 4:**
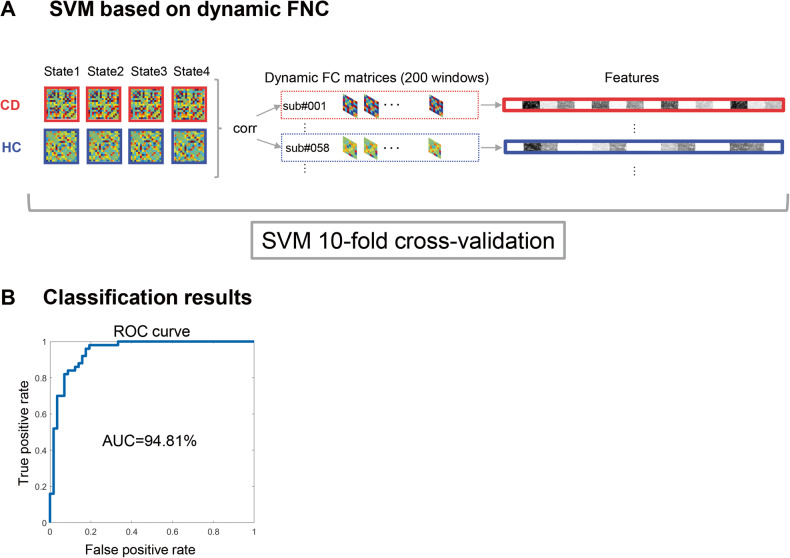
The results of classification. **A** An overview of classification approach. We first extracted the averaged FNC pattern for each state for each group. Then we performed Pearson correlation between the FNC in each window and the FNC pattern in all states among all groups. These procedures ended up with 8 averaged features for each participant. **B** Receiver Operating Characteristic (ROC) curves for classification. SVM support vector machine, AUC area under the curve.

The support vector machine (SVM) based on dynamic FNC approach (Fig. 4A, details see Method) showed classification accuracy of 84.76% for CD patients, 88.98% for healthy controls (Fig. 4B). The classification scores were evaluated using a receiver operating characteristic (ROC) curve aiming to visualize the performance of the classifier. The classification results may further indicate that the dynamic functional connectivity pattern within these three networks would be the potential biomarker of individuals with excessive higher cortisol level.

## Discussion

In the current study, we adopted independent component analysis (ICA) and dynamic functional connectivity (FNC) approaches to reveal the difference in dynamic FNC within DMN, SN, and CEN networks between CD patients and healthy controls. Using clustering algorithm, we defined four reoccurring FNC states during resting-state scanning. Wherein State 1 and State 4 exhibited significant differences between healthy control and CD patients. Patients generally showed more dwell time in State 1 but less in State 4 than healthy controls. Specifically, in State 1, the CD patients showed weaker connections within DMN, as well as weaker intra-network connectivity between DMN and CEN, SN and CEN than healthy controls. In State 4, connections between DMN and SN, DMN and CEN showed weaker connection in CD patients than in healthy participants. Further correlation and mediation analyses showed that the dwell time in State 1 significantly negatively correlated with cognitive performance. While dwell time in State 4, as well as the connections between DMN and SN in State 4, were found to positively correlate with cognitive performance, and negatively associated with depression and anxiety symptoms. Both states were associated with physiological indices including cortisol, ACTH and 24-hour UFC. Importantly, results from mediation analysis indicated the difference between CD patients and healthy controls on dwell time in State 1 and State 4 can be used to explain their cognitive performance difference. Intriguingly, adopting support vector machine algorithm based on dynamic FNC within DMN, SN and CEN network generally showed ideal classification accuracy for CD patients and healthy controls. These findings begin to delineate the dynamic properties of the three brain networks, which are critical for cognitive and neuropsychiatric, and open new avenues for understanding and explaining the impaired cognitive performance and psychiatric symptoms induced by Cushing’s disease.

We found two distinct functional connectivity states across two groups. State 1 can be characterized as having weak connections among the three networks, while State 4 showed relatively strong inter-network and intra-network connections. We observed that in patients with Cushing’s disease, State 1 occurred more often, while State 4 occurred less than in healthy controls. These results help to confirm CD patients’ weaker connections within DMN, SN, and CEN. Previous studies identified that white matter integrity was generally decreased throughout the whole brain rather than just on individual fasciculus [45–47]. One possible explanation is that the extensive decline in white matter structural integrity leads to the decreased connectivity of the three networks, which are critical for the cognitive-affective process. We found that in State 1, CD patients showed decreased local synchronization (i.e., within network connectivity) of DMN, and weak inter-network connections between CEN and DMN, CEN and SN. The DMN’s integrity appears crucial for cognitive performance. For example, patients with Alzheimer’s disease showed decreased connectivity within DMN [48]. Since dwell time of State 1 was negatively correlated with MoCA and mediated the group differences on MoCA. We may infer that cognitive deficit may be due to that CD patients engaged more time in State 1 with weak connections of DMN.

Interestingly, the more dwell time in State 4, the less anxiety and depression symptoms individuals would have. Moreover, our further analyses found that connections between DMN and SN during State 4 would also negatively affect anxiety and depression. And the CD patients had weaker DMN-SN connections than healthy controls in this state. In line with previous studies, effective connectivity from DMN to SN was lower in major depression disorders compared to healthy controls when processing negative information [49]. And the inter-network connections between the SN and DMN were inversely associated with trait anxiety levels [50]. Therefore, the time engaged in State 4 and the weak inter-network connectivity between SN and DMN may contribute to psychopathological symptoms in CD patients.

Dynamic functional connectivity provides time-varying rather than static features over time [11], and it is more effective to capture various aspects of brain connectivity. The dFNC approach has obvious advantages for classification purposes [35]. For example, previous research showed high classification accuracy for psychiatric diseases such as schizophrenia [51], and bipolar [35]. In our study, the SVM based on dynamic functional connectivity features within DMN, SN and CEN showed high classification accuracy for CD patients and healthy controls, which may indicate that the dynamic properties in these three networks would be potential biomarkers for individuals with excessive higher cortisol level.

The long-term remitted CD (LTRCD)-patients still suffered from cognitive impairments and emotional symptoms such as anxiety and depression, even though their cortisol levels back to normal after the removal of the adenoma [2, 52, 53]. We revealed that the dynamic features in DMN, SN, and CEN correlate with depression and anxiety symptoms in CD patients and are strongly associated with cognitive performance. Our findings may contribute to developing further neuro-modulation targets to help CD patients improve cognitive ability and mental health.

Several limitations of the present study should be mentioned. First, Cushing’s disease is rare, and it is more common in women [1, 3]. We only showed results based on a female sample (healthy controls were all female). Therefore, our conclusion may not be adaptive for the male population. Second, some research suggested that the dynamic functional connectivity analyses should be performed in resting state acquisitions of at least ten minutes [54]. The length of current resting-state scan was eight minutes, although many previous studies studied dynamic FNC based on resting-state data in eight minutes or even less [13, 20, 51], further studies should consider longer scanning to capture more dynamic spontaneous features. Thirdly, our results revealed that cortisol concentrations were significantly associated with dwell time in State 1 and 4 but were not correlated with inter-network or intra-network connections. Human cortisol secretion has apparent circadian rhythmicity [55], but our resting state acquisitions were not collected multiple times. Our conclusions may not be informative to understand the relationships between dynamic functional connections and dynamic cortisol levels.

In conclusion, our study delineates the differences in dynamic properties between CD patients and healthy participants. It unravels its associations with cognitive deficits, impaired affective processes, and physiological indices in CD patients. We believe the temporal dynamics of functional connectivity within the three crucial cognitive and affective brain networks could be a promising imaging biomarker to monitor cognitive changes and psychiatric symptoms in Cushing’s disease.

### Supplementary information

Supporting information

## Data Availability

All datasets are available on figshare. https://figshare.com/projects/Dynamic_functional_connectivity_changes_associated_with_psychiatric_traits_and_cognitive_deficits_in_Cushing_s_disease/170343.

## Code availability

All code used for all analyses and plots are publicly available on GitHub at https://github.com/psywalkeryanxy/paper_CD_ICA.
