# Supplementary material for: Dynamic functional connectivity changes associated with psychiatric traits and cognitive deficits in Cushing’s disease
Source: Transl Psychiatry. 2023 Oct 5;13:308. doi: 10.1038/s41398-023-02615-y (PMC10556150; doi:10.1038/s41398-023-02615-y)
Supplement: Supplementary file 1 — Supporting information [file 41398_2023_2615_MOESM1_ESM.docx]

**Supporting information**

**Table S1. Fractional windows (%) of dynamic FNC states between CD patients and healthy controls.**

|  | CD (mean±SD) | HC (mean±SD) | t (CD vs. HC) | p-value |
| --- | --- | --- | --- | --- |
| State 1 | 42.080(28.827) | 33.640(23.512) | 1.667 | 0.098 |
| State 2 | 22.780(28.868) | 13.040(18.900) | 2.087 | 0.039 |
| State 3 | 15.910(18.769) | 21.180(18.644) | -1.453 | 0.149 |
| State 4 | 19.230(20.802) | 32.140(24.591) | -2.909 | **0.004** |

CD = patients with Cushing’s disease; HC = Healthy controls. Significant results which were survived after FDR (p<0.05) correction were reported in bold type.
